# Realistic Visual Acuity and Patient Satisfaction After Simultaneous Bilateral Tecnis Eyhance DIB00 Monofocal Plus Lens Implantation During a 12-Month Follow-Up Period in a Single-Center Study

**DOI:** 10.3390/medicina62071288

**Published:** 2026-07-03

**Authors:** Agnieszka Nowosielska, Grzegorz Rotuski

**Affiliations:** Warsaw Eye Hospital, Wolska 165/U7, 01-258 Warsaw, Poland

**Keywords:** Tecnis Eyhance, simultaneous bilateral lens removal, mesopic vision, near vision, quality of vision, monofocal plus intraocular implant

## Abstract

*Background and Objectives*: The purpose of this study is to present the clinical results over a 12-month follow-up period in patients after simultaneous bilateral crystalline lens removal with implantation of a Tecnis Eyhance DIB00 monofocal plus lens. *Materials and Methods*: This is a single-center, 12-month, retrospective study involving 214 eyes of 107 patients operated on between 2021 and 2023. Measurements included uncorrected and best-corrected visual acuity at 30 cm, 70 cm, and distance vision under fixed lighting conditions, instead of focusing on patient-dependent factors, such as pupil size, which varies individually based on sympathetic and parasympathetic nervous systems balance, simulating visual performance in real-world conditions. Contrast sensitivity was also assessed at 6 and 12 months postoperatively. Patients filled out a questionnaire on quality of life after surgery. *Results*: Uncorrected and best-corrected visual acuity were, respectively, for a 30 cm distance UCVA 0.76/BCVA 0.18 LogMAR; for a 70 cm distance UCVA 0.03/BCVA −0.036 LogMAR; and for distance vision UCVA 0.05/BCVA 0.02. None of the patients reported adverse phenomena such as halo, glare, contrast decrease, or other phenomena after surgery. The average rating of patient satisfaction assessed in a special questionnaire was 8.54 out of 10 points. *Conclusions*: The Tecnis Eyhance DIB00 lens implanted during bilateral cataract surgery produced good results in terms of uncorrected visual acuity under various lighting conditions, which in our population was better than predicted by the lens manufacturer. Through the 12-month observation period, postoperative refractive values remained stable, with no deterioration of vision.

## 1. Introduction

Lens removal surgery, whether for cataracts or to correct a refractive defect, is the most commonly performed surgical procedure in the world. Currently, any lens removal is a refractive surgery [1]. The growing expectations of patients to see without glasses at all distances after the surgery have placed a demand on the surgeon that, in addition to a perfectly performed surgical procedure, the intraocular implant should be selected to suit the patient’s needs, and the power should be calculated in such a way that no refractive defect remains after surgery [2,3,4].

For today’s patient, ideal distance vision is no longer enough. The change in lifestyle that has taken place recently has necessitated the construction of lenses that allow one to see at intermediate and/or near distances. The fact that younger and younger patients are opting for lens replacement imposes an additional task on the ophthalmic team to ensure that the procedures are performed quickly and efficiently, and that the patient may complete visual rehabilitation as soon as possible. Multifocal lenses and extended depth of focus (EDOF) lenses have appeared in the product portfolios of companies. However, they have limitations, especially when it comes to undesirable optical phenomena such as halo and glare [2,5].

The Tecnis Eyhance DIB00 lens from Johnson & Johnson Vision, Irvine, CA, USA, is a lens with an innovative design that provides vision at intermediate distances. It was approved for the U.S. market by the FDA (Food and Drug Administration) in 2019. It has been available in Poland since 2020. Tecnis Eyhance is a monofocal lens with increased depth of field. This was achieved thanks to the special design of its front surface, which, thanks to its asphericity that gives gradual defocus to the level of one diopter, allows vision at intermediate distances. According to the manufacturer’s declaration, the Tecnis Eyhance lens provides good distance vision and intermediate vision at 70 cm [5,6,7,8,9]. As this lens retains the design of a monofocal lens, there should be no dysphotopsias after implantation.

The time of the COVID-19 pandemic in 2020–2023 imposed on medical professionals the need to maximize the number of medical procedures with as few medical visits as possible to limit the time of exposure to potential infection. It is then that at the Warsaw Eye Hospital we began to perform lens removal procedures simultaneously in both eyes, which has since become our routine procedure.

## 2. Methods

The medical records of 214 eyes of 107 patients operated on at the Warsaw Eye Hospital between 2021 and 2023 were retrospectively analyzed. All patients underwent bilateral, simultaneous natural lens replacement surgery with Johnson & Johnson’s Tecnis Eyhance DIB00 implant. The study was conducted in accordance with the guidelines of the Declaration of Helsinki and approved by the Bioethics Committee at the Warsaw Medical University, AKBE/148/2023, approval date: 8 May 2023.

Inclusion criteria:

- Age over 40 years.
- Baseline pupil width was not evaluated on the assumption that in the case of a monofocal lens implant such as the Tecnis Eyhance lens, baseline pupil width should not be important in qualification for this type of implant.
- Patients who underwent uncomplicated phacoemulsification of senile cataract with implantation of Johnson & Johnson’s Tecnis Eyhance DIB00 monofocal plus lens.
- Patients with an eyeball without significant pathology as confirmed by OCT (optical coherent tomography) of the macula and optic nerve disk examination.

Exclusion criteria:

- Patients who have previously undergone vision correction surgery.
- Concurrent corneal diseases such as corneal cone and corneal damage.
- Low number of corneal endothelial cells below the threshold of 1000 cells per mm^2^.
- Instability of the lens ligament apparatus regardless of cause.
- Corneal astigmatism higher than 1D, which, according to the authors, requires the implantation of a toric lens.
- Complications of cataract removal surgery related to anterior or posterior capsular tear.
- Cataract removal surgery combined with vitrectomy.
- Unstable glaucoma undergoing treatment with drugs resulting in optic nerve damage.
- Concurrent retinal diseases such as epiretinal membrane, macular hole, macular drusen, and others.
- Any previous eye surgery.

Prior to surgery, all patients were tested to check visual acuity, without and with correction for distance and near vision, intraocular pressure was measured, and also a standard anterior and posterior segment examination was performed after the administration of pupil dilating drops. Corneal topography examinations were performed on a Pentacam camera (Oculus, Wetzlar, Germany), and an intraocular lens power calculation was performed. The method of choice was the Barrett fourth-generation formula.

The surgical procedure in each patient was performed simultaneously on the right and left eyes. The procedure was performed by the same experienced surgeon using an EVA from Dorc (now The Zeiss Company, Oberkochen, Germany). The main port selected had a diameter of 1.8 mm. All patients had a transparent dressing applied to the right and left eye after the surgery, allowing for a certain degree of vision.

Postoperative examination was performed 1 day after the surgery, 7 days after the surgery, and 28 days after the surgery. During these follow-up examinations, the condition of the eyeball after surgery was monitored, and the occurrence of potential postoperative complications (corneal damage, intraocular inflammation, retinal detachment, etc.) was checked. The refraction and quality of vision examination, which was used to statistically compile the results, was carried out after 6 and 12 months following the surgery of simultaneous lens removal in the right and left eyes. It included: a standard optometric and ophthalmic examination (intraocular pressure, anterior segment, and posterior segment). The optometric examination included measurement of contrast sensitivity on contrast testing ETDRS boards (Precision Vision, Woodstock, IL, USA), postoperative refractive examination with testing of vision with and without correction for each eye individually at different distances of 30 cm, 70 cm, and at far distance under varying lighting conditions. A brightness of 390.4 LUX was determined as good lighting conditions and 11.0 LUX as low lighting conditions, measured in a fully dark room with the only source of lighting being regulated by a luxometer (URCERI MT-92H Light Meter, Shenzhen Flus Technology Co., Ltd., Shenzhen, China).

All optometric procedures were performed in a standardized optometric office. The results of visual acuity testing were recorded on the LogMAR scale. Information on postoperative refraction and postoperative keratometry was collected. Pre- and post-operative refraction was presented in the form of spherical equivalent, measured for each eye separately.

Statistical analysis was performed using the IBM SPSS Statistics 25 package:

- To check whether the compared groups of people are equal in number, the chi-square test was used.
- To check whether there are statistically significant differences between two measurements within the same group of people, the Wilcoxon test was used. When measurements were made across more than two variables within the same group of people, the Friedman test was used. Non-parametric equivalents of statistical tests were used due to significant disturbances in the normality of the distribution.
- Spearman correlation analysis made it possible to check whether there was a statistically significant relationship between the analyzed variables.
- The following descriptive statistics were used in the statistical analysis of the results: mean, standard deviation, median, minimum maximum, first and third quartile.

Measurements were performed for both eyes separately. To account for inter-eye correlation, we analyzed the collected data per patient as a unit of measurement. Linear Mixed-Effects Models (LMM) were used to correct for multiple comparisons. Normality of the data was assessed both visually using box and Q-Q plots with histograms and formally using the Shapiro–Wilk test. Bonferroni Correction and the Benjamini–Hochberg Procedure were used to reduce the risk of false positive and false negative results.

## 3. Results

The study included 107 patients, 52 (48.6%) of whom were female (χ^2^(1) = 0.08; *p* = 0.77). The average age of the patients was 65 years (Table 1). All patients underwent, without complications, a bilateral, simultaneous cataract removal and implantation of Johnson & Johnson’s Tecnis Eyhance DIB00 lens.

In all patients entered in the study, the power of the intraocular lens was calculated to achieve emmetropic vision after surgery. No form of monovision was used. The formula standardly used to calculate intraocular lens power was the Barrett fourth-generation formula. The length of the eyeballs in the study population varied between 20.59 mm and 27.15 mm, with an average eyeball length of 23.63 mm. The average postoperative refraction measured as spherical equivalent was −0.35 D and did not change over the 12-month follow-up period (mean: −0.35047 D, range: −1.25 D; +0.75 D, median: −0.25 D, SD: 0.412792).

The uncorrected visual acuity for a distance of 30 cm was similar for poor and good lighting conditions and was 0.76/0.78 LogMar, respectively. For corrected BCVA visual acuity, the results under poorer lighting conditions, i.e., with a wider pupil, were worse at 0.23 LogMar, and for better lighting conditions and a narrower pupil, they were 0.29 LogMar. This difference was statistically significant (Z = 3.36, *p* = 0.001). The visual acuity results for a distance of 30 cm are shown in Table 2.

At a distance of 70 cm, both UCVA and BCVA visual acuity appeared to be better under poorer lighting conditions. UCVA was 0.03 and −0.03 LogMar for better and worse lighting conditions, respectively. The best corrected visual acuity from this distance was −0.04 LogMAR for better lighting conditions and −0.01 LogMar for poorer lighting conditions. These results are statistically significant (Z = 3.36; *p* = 0.001), as shown in Table 3.

**Figure 1:**
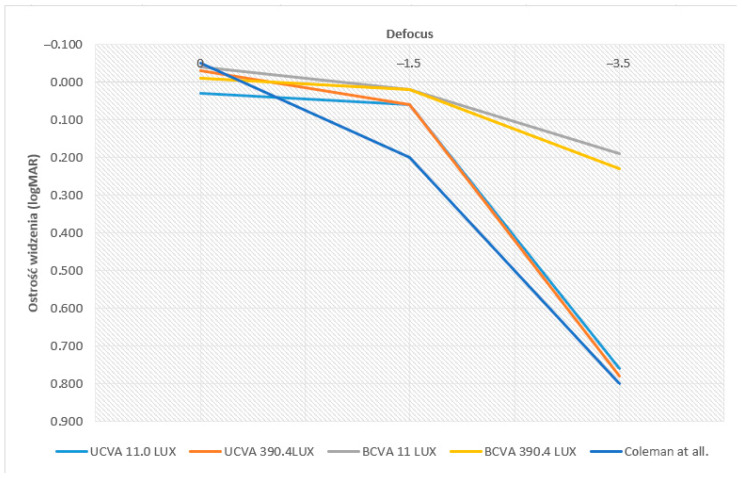
Defocus curve for corrected and uncorrected visual acuity of Tecnis Eyhance lenses and the Coleman at al study [10].

The defocus curve obtained for our material for the Tecnis Eyhance lens overlaps with the defocus curves for EDOF lenses reported, for example, by Coleman [10]. The defocus curve is shown in Figure 1, presenting visual acuity for far vs. 70 cm (−1.5 D) and 30 cm (−3.5 D) reading distances in different lighting conditions.

For distance acuity, the visual acuity of both UCVA and BCVA in the study group under varying lighting conditions was the same. The results are shown in Table 4. This shows that after implantation of the Tecnis Eyhance DIB00 lens excellent acuity for distance vision was achieved regardless of the prevailing lighting conditions.

The achieved visual acuity at all distances was stable during the 12-month follow-up period. There was no case of posterior bag opacification that would require a capsulotomy surgery. In the postoperative period, there were no significant postoperative complications such as intraocular inflammation, retinal detachment, implant dislocation, etc. Patient satisfaction was assessed on the basis of the Refractive Cataract Surgery Survey (RCSS) [10]. The maximum number of points possible in the questionnaire was 10.00 on a scale of 1–10, where patients mark their satisfaction with vision without glasses at far, intermediate, and near distances, glasses independence after surgery, whether the expectations were met, and the service quality provided by the surgeon. The average number of points granted by all patients in the survey was 8.54.

Patients did not report the appearance of any optical phenomena such as halos or glares after the lens removal surgery. They did not associate the bilateral simultaneous lens replacement procedure with any unpleasant sensations. They did not describe the bilateral procedure as inconvenient. They usually emphasized a greater ease of adaptation to their new seeing abilities and a significant reduction in postoperative rehabilitation time, as well as less inconvenience for third parties who took care of them during the postoperative period.

## 4. Discussion

Johnson & Johnson’s Tecnis Eyhance implants have been new to the market for several years and have proven their quality in numerous research studies. The original approved implants were ICB00 lenses, implanted with the use of an injector. However, the updated version marked with the DIB00 symbol has been introduced, offered in a preloaded system, i.e., they are already placed in a cartridge. This new system is interesting for several reasons. Placing the implant in a cartridge beforehand reduces the time of surgery and makes it easier to insert into the eye. The possibility of accidental damage by the surgeon to the haptic part of the implant during implantation is therefore minimized. The preloaded system enables the surgeon to insert an implant of virtually any power through a 2.2 mm cut rather than the 2.4 mm cut needed for the top of the injector. This lowers the likelihood of postoperative astigmatism, which is also relevant when assessing for postoperative halo, glare and starburst signs.

The term monofocal plus relates to the structure of the IOL providing typical sharp vision for target distance, but with a slightly modified central zone to give an optical addition for closer distances, just enough to avoid dysphotopsias [11]. Due to its safety profile, it can be considered even in eyes with low vision due to amblyopia or other ocular comorbidities. Tecnis Eyhance is an IOL model made of hydrophobic acrylic material, with a refractive index of 1.47, non-diffractive, and aspheric in design, which reduces optical aberrations. It has not met the American National Standards Institute (ANSI) criteria for EDOF, but is superior to monofocal IOL due to its higher-order aspheric anterior surface that creates a continuous power progression from periphery to center [12]. Analyzing defocus curves of optical spectra of IOLs is helpful but does not necessarily reflect their performance on the quality of vision [13]. The key term is to tailor the best compromise for the patient’s lifestyle. Exaggerated expectations should restrain the choice of non-monofocal IOL. The psychological profile of patients can often correlate with their tolerance of dysphotopsias, as is the case for vitreous floaters.

Sometimes monocular cataract surgery with a correct refractive outcome of a premium IOL can help decide about the other eye correction to fit the patient’s needs and inconveniences. For instance, EDOF inserted in one eye that is not tolerated by the patient due to halos/glare might incite the patient to choose a monofocal alternative for the fellow eye to minimize adverse optical effects and then attempt waiting for neuroadaptation to avoid the necessity of IOL exchange [14]. Moreover, these adversities, along with no benefit, may be encountered in patients with ocular comorbidities, such as glaucoma or retinal diseases [15,16]. When choosing the power of monofocal plus lenses intended for both eyes simultaneously, the goal is oftentimes to give some range of vision. Therefore, the dominant eye is more directed towards emmetropia, while the non-dominant one is aimed more at a slight myopic refraction, typically between −0.50 and −0.75 diopters. However, this choice has to be based on a preoperative assessment of anisometropic tolerance, along with a discussion of the patient’s profession and hobbies, to fit the needs to the greatest extent possible.

In our present study, we focused on measuring visual acuity under varying lighting conditions to mimic those encountered in real life. We evaluated uncorrected visual acuity in patients implanted with Tecnis Eyhance DIB00 lenses, focusing on lighting conditions rather than on different pupil widths. We took a different approach from most authors, who usually take pupil width as a starting point [17]. This was dictated by the observation that lighting conditions will change in everyday life, while pupil width varies individually regardless of environmental conditions; for instance, pupils are smaller in the elderly due to reduced activity of the sympathetic system. Therefore, if we wish to study the visual acuity of the patients in real-life conditions, we should concentrate on adjusting lighting conditions, which is much easier to achieve than aiming for a specific pupil width induced pharmacologically or surgically.

Light conditions also affect the vision quality. If the image is distorted by the multifocal lens, the amount of light is reduced for each perceived distance. Hence, in photopic conditions, the deficit is not noted due to abundant light, but a difference may be noticeable and bothersome in dim light conditions. Previous research found confounding results regarding contrastometry in varied combinations of IOLs and lighting conditions [18,19]. According to our study, lighting conditions have no effect on distance visual acuity [20].

Mesopic conditions encompass low light around 0.01–3 cd/m^2^ (equivalent to 0.01–50 LUX), when both rods and cones are active and enable one to see colors [21,22]. Scotopic conditions, on the other hand, occur in darkness with light levels below 0.01 LUX, surroundings being perceived solely by rods. The latter is generally happening very occasionally, mostly at night awakenings with the choice of not lighting the room or cutting electricity on the way to the bathroom, and other unique scenarios such as cave explorations, etc. In these situations, the visual acuity potential is much lower in all individuals, and reliance focuses on other senses for spatial orientation. Since the fovea centralis is almost exclusively constituted of cones, which do not function in a scotopic environment—the mean VA of the perifoveal region around 0.2 Snellen and decreasing even more towards the periphery—these conditions are always demanding, and due to their relatively rare occurrence throughout the 24 h, this was not tested due to the mostly scientific and not really practical purpose.

Particularly in autumn–winter time, when days are shorter, there is a frequent need for additional lighting, and the way to and from work is often in dim light environments; the amount of light transmitted through the IOL, along with different refraction angles on transition zones of the IOL, can be more bothersome for patients. Scotopic conditions can reduce the quality of vision, mostly when lighting in a given space is uneven, so when a light source shines from focal points on a dark background. This occurs mostly while driving, with the head-up display being more prominent in regard to darkness after dusk, along with streetlights and other cars’ headlights. But also in passionate readers, visual performance may be more wearisome in dim light. In the last case, the suggestion is first to increase the amount of lighting, e.g., ceiling + desk bulb, which is harder to achieve for drivers.

With the DIB00 implants, it is possible to achieve distance acuity comparable to or higher than that achieved with the implantation of classic monofocal lenses Tecnis ZCBOO [23]. In our material, despite the residual refractive error that remained in the study group at −0.35 D, excellent uncorrected distance acuity was achieved, regardless of lighting conditions. This is consistent with the results that Mrugacz et al. received. In their work, the average residual defect was −0.375 D, and, despite this, uncorrected visual acuity remained high [24]. The uncorrected visual acuity for intermediate distances described in our material appears to be similar to that cited by other authors.

However, there is great variation among authors in the literature as to what distance is considered intermediate. Depending on the work, the intermediate distance is 65 cm in Gigon or Affarth, 66 cm in Menucci, or 80 cm in Yongzes. It is, therefore, not surprising that the visual acuities obtained by these authors are slightly different [7,16,25,26,27]. However, it cannot be assumed that visual acuity at intermediate distances obtained as a result of an approximate calculation based on the measurements cited in the above-mentioned publications will correspond to visual acuity at intermediate distances in the real-life conditions studied in our work [28]. The distances at which the visual acuity test was performed and the pupil width adopted in the various publications are shown in Table 5. Based on observations of the distances at which vital activities are performed, it seems that the intermediate distance of 70 cm adopted is most adequate. This is also the average distance quoted by other authors.

Intermediate vision requirements depend on the task performed. Nowadays, most people will need a computer, either for pleasure or work purposes, the latter being more prevalent with the constant increase in remote work settings, and distance from the eyes depends on the dimensions of the room and the desk, among others, which may be challenging to modify. Cooking, playing musical instruments, or dentistry and surgery are other tasks with variable distances depending on height relativity. These arguments may speak in favor of aiming at some degree of monovision for patients to give them as much glass independence as possible due to no unified intermediate distance in everyday life.

The case is similar with regard to the study of visual acuity at near distances. Here, too, the authors give varying distances. For Gigon, the near distance is 35 cm; for Mencucci and Choi, it is 40 cm. Some authors, acting on the assumption that Tecnis Eyhance does not provide for near vision, did not test near visual acuity at all, for example, Auffarth [7,16,25,26,27]. In fact, the near visual acuity distance depends on the position of the patient, which is not constant depending on the furniture used, including changes made to get more comfortable. Aside from typical text and picture reading, either on paper or digital devices, some professions and hobbies require very detailed visual accuracy, such as sewing, knitting, crafting, jewelry, watchmaking, and electronic and laboratory technicians. In some publications, better visual acuity was obtained by using some form of monovision. In our work, very importantly, no form of monovision was used, and all eyes were calculated before surgery to obtain normal vision after surgery. Thus, we examined near visual acuity in eyes with emmetropia.

We further proved in our work a stable post-surgical refractive effect after a period of 12 months. The unique measurements we made in our work showed that visual acuity at intermediate distances after Tecnis Eyhance DIB00 lens implantation is better in poorer lighting conditions, while at a distance of 30 cm, it does not deteriorate as lighting conditions worsen. This is an important feature of the implant from a practical point of view, as it provides patients with good vision in deteriorating external illumination. Patients in our study did not experience halo or glare, which would be expected given the lens design, nor did they have a sense of deteriorated contrast.

The simultaneous bilateral cataract surgery still poses controversies among ophthalmic surgeons. The lens removal procedure performed bilaterally has its advantages in terms of time and cost savings and reduced inconvenience for patients and their families. After a bilateral lens removal, visual rehabilitation is faster, and the possibility of selecting nearsighted spectacle correction for both eyes at the same time allows for a faster return to both professional activities and hobbies [29].

Nonetheless, the issue might often be restrictions from national health insurance in some countries, like Poland, that better finance two separate single-eye procedures than both eyes done during one hospitalization in public institutions. This is contradictory to the objective fact that, taking into account all pre- and postoperative preparations and checks, the bilateral approach is more time- and cost-effective. Therefore, we postulate that simultaneous binocular cataract surgery should be the standard of care, with monocular as an option of exclusion [30]. In the hands of modestly experienced surgeons, this approach might not be so straightforward, with the necessity for delayed second-eye cataract surgery on the occurrence of more serious complications such as posterior capsule rupture or lens drop, which may also happen at any time.

The positive aspect of this approach is certainly the possibility to recalculate the implant power of the second eye in case of a refractive surprise in the first eye. The Barrett Universal, being currently the reference formula in case of divergent results in the others, may not always be right. The ESCRS and ACRS expert guidelines also suggest taking EVO, Hill-RBF, and Cooke K6 into consideration due to their highest accuracy in comparative studies [31]. In case of a different subjective refraction postoperatively than intended, the choice ranges between external optical correction, which is not optimal from the quality of life point of view, performing an adjustment procedure on the cornea, or, in the worst-case scenario, IOL explantation and replacement with a new power. Keeping a slight inter-eye refractive difference can help give more depth of focus, but some individuals might not adapt easily.

The most worrying, yet rarely occurring, complication in bilateral simultaneous cataract surgery is sight-threatening endophthalmitis that could spread through the use of poorly sanitized equipment used in both eyes in the same session. Multi-step protocols to check the quality of the operating room’s preparation should be the most emphasized counteractive measure, along with a thorough explanation of the prophylaxis to the patient [32]. We did not record a single case of intraocular inflammation or other serious event.

A sudden change in binocular vision, particularly if the cataract was denser or the refractive change was important, might cause temporary strabismus, which should resolve in the early postoperative period [33]. The risk is increased in patients with a positive strabismus medical history, but it should not be a contraindication to binocular surgery. They should be warned about intermittent diplopia or other postoperative visual disturbances, but like any patient, the first 2–6 weeks (depending on individual needs) should be spent on recovery.

Pain and discomfort associated with the surgery are another factor of consideration [34]. One argument is the possibility of avoiding double trauma upon binocular simultaneous operation. This depends on individual tolerance to pain, of course, but also the incision site in relation to corneal nerve density and their later regeneration. A registration of the corneal innervation map could be made preoperatively, for example, through confocal microscopy [35].

When it comes to the elderly, the shorter time of the procedure will help them reposition or regain breathing. If the vision is low due to neuroretinal impairment, or patients are not mobile, especially in a statistically short expected life duration, the rational choice would be monocular surgery (some peripheral vision gain and less blurriness). Obviously, when the cataract is uneven, for example, after eye trauma or steroid-induced PSC, the fellow eye should not be operated on without an expected benefit at the moment of surgery, meaning that anticipating cataract formation should not be an indication.

Some patients will also give their opinion if they wish to have one eye done at a time, and this is binding for the surgical decision.

The limitations of our study consisted, undoubtedly, of its retrospective nature and lack of randomization. The strengths of our study are the large number of patients studied, the long follow-up period of 12 months, the study of near visual acuity under varying lighting conditions, which is unique in studies, and that we studied the implant in situ with preloaded DIB00.

## 5. Conclusions

Monofocal plus implants can provide functional uncorrected visual acuity at most distances in patients with a higher risk of adverse effects for “premium” intraocular lenses (EDOF, multifocals). The visual performance remains non-inferior in mesopic conditions, with good visual effects persisting in varying light conditions. Furthermore, simultaneous bilateral natural lens removal and IOL implantation may lead to a more efficient visual recovery without significant risks in relation to the typically performed unilateral approach. In order to prevent a refractive surprise, several calculating formulas should be used beforehand, and in case of discrepancies between results, consider doing one eye at a time.

## Figures and Tables

**Table 1:** Descriptive statistics regarding the age of the people under study.

Group	M	Me	SD	Min	Max	Q1	Q3
Tecnis Eyhance	65	67	12	40	90	56	74

**Table 2:** Descriptive statistics for UCVA and BCVA measured in LogMar values at a distance of 30 cm for lighting of 11.0 LUX and 390.4 LUX.

Group		UCVA 11.0 LUX 30 cm Distance	UCVA 390.4 LUX 30 cm Distance	BCVA 11.0 LUX 30 cm Distance	BCVA 390.4 mm 30 cm	Statistical Test Result	
						UCVA	BCVA
Tecnis Eyhance	M	0.76	0.78	0.19	0.23	Z = 1.34; *p* = 0.18	Z = 3.36; *p* = 0.001
	SD	0.15	0.13	0.08	0.08		
	Min	0.53	0.36	0	0		
	Max	1.2	1.14	0.26	0.36		
	Q1	0.62	0.66	0.13	0.13		
	Me	0.84	0.84	0.13	0.26		
	Q3	0.84	0.84	0.26	0.26		

**Table 3:** Descriptive statistics for UCVA and BCVA measured in LogMar values at a distance of 70 cm for lighting of 11.0 LUX and 390.4 LUX.

Group		UCVA 11.0 LUX mm 70 cm	UCVA 390.4 LUX mm 70 cm	BCVA 11.0 LUX mm 70 cm	BCVA 390.4 LUX mm 70 cm	Statistical Test Result	
						UCVA	BCVA
Tecnis Eyhance	M	0.03	−0.03	−0.04	−0.01	Z = 0.29; *p* = 0.77	Z = 2.87; *p* = 0.004
	SD	0.24	0.14	0.05	0.15		
	Min	−0.1	−0.23	−0.1	−0.23		
	Max	0.9	0.25	0	0.25		
	Q1	−0.1	−0.1	−0.1	−0.1		
	Me	0	−0.1	0	−0.1		
	Q3	0	0.17	0	0.17		

**Table 4:** Descriptive statistics for UCVA and BCVA measured in LogMar values for varying lighting conditions when testing far distance acuity.

Group		UCVA 11.0 LUX Distance	UCVA 390.4 LUX Distance	BCVA 11.0 LUX Distance	BCVA 390.4 LUX Distance	Statistical Test Result	
						UCVA	BCVA
Tecnis Eyhance	M	0.06	0.06	0.02	0.02	Z = 1; *p* = 0.32	Z = 0; *p* = 1
	SD	0.11	0.11	0.06	0.06		
	Min	0	0	0	0		
	Max	0.7	0.7	0.2	0.2		
	Q1	0	0	0	0		
	Me	0	0	0	0		
	Q3	0.1	0.1	0	0		

**Table 5:** Comparison of visual acuity under varying lighting conditions.

Author	Implant Model	Observation Time in Months	Lighting Conditions	Intermediate Distance [cm]	Near Distance [cm]	Near Vision Acuity Without Correction LogMar	Intermediate Vision Acuity Without Correction LogMar	Distance Vision Acuity Without Correction LogMar
Nowosielska et al.	DIB00	12	Scotopic	70	30	0.76	0.03	0.06
			Photopic	70	30	0.78	−0.03	0.06
Gigon et al. [28]	ICB00	1–12	Photopic	65	35	0.3	0.0	0.03
Mencucci et al. [25]	ICB00	3	Photopic	65	40	0.475 ± 0.119	0.283 ± 0.096	0.041 ± 0.042
Auffarth et al. [7]	ICB00	6	Photopic	65	-	-	−0.02 ± 0.01	0.16 ± 0.02
Choi Hyun et al. [26]	ICB00	3	Mesopic	66	40	-	0.03 ± 0.05	0.05 ± 0.05
Yangzes et al. [27]	ICB00	3	Photopic	80	40	0.43 (0.13)	0.10 (0.13)	0.11 (0.13)

## Data Availability

The original contributions presented in this study are included in the article. Further inquiries can be directed to the corresponding author.
